# The concordance of treatment decision guided by OncotypeDX and the PREDICT tool in real‐world early‐stage breast cancer

**DOI:** 10.1002/cam4.3088

**Published:** 2020-05-06

**Authors:** Daniel A. Goldstein, Chen Mayer, Tzippy Shochat, Daniel Reinhorn, Assaf Moore, Michal Sarfaty, Rinat Yerushalmi, Hadar Goldvaser

**Affiliations:** ^1^ Davidoff Cancer Center Beilinson Hospital Rabin Medical Center Petach‐Tikva Israel; ^2^ Sackler Faculty of Medicine Tel Aviv University Tel Aviv Israel; ^3^ Department of Health Policy and Management Gillings School of Global Public Health University of North Carolina at Chapel Hill Chapel Hill NC USA; ^4^ Department of Pathology Sheba Medical Center Ramat Gan Israel; ^5^ Statistical Consulting Unit Beilinson Hospital Rabin Medical Center Petach‐Tikva Israel

**Keywords:** adjuvant, breast cancer, genomic assays, oncotype, predict tool

## Abstract

**Background:**

Decision‐making regarding adjuvant chemotherapy for early‐stage breast cancer can be guided by genomic assays such as OncotypeDX. The concordance of expected clinical decisions guided by OncotypeDX and prognostication online tools such as PREDICT is unknown.

**Methods:**

We performed a retrospective single‐center cohort study comprising all women with estrogen receptor (ER) positive, human epidermal growth factor receptor 2 (HER2) negative, node negative disease, whose tumors were sent for OncotypeDX analysis. Expected decision on adjuvant chemotherapy was evaluated using OncotypeDX and using PREDICT. The concordance between these two tools was calculated. The impact on concordance of prespecified features was assessed, including age, tumor size, intensity of ER and progesterone receptor (PR), grade, Ki67 and perineural and lymphovascular invasion.

**Results:**

A total of 445 women were included. Overall concordance was 75% (*K* = 0.284). The concordance was significantly higher for grade 1 disease compared to grade 2‐3 (93% vs 72%, *P* < .001), tumor ≤ 1 cm compared to >1 cm (85% vs 72%, *P* = .009), PR positive compared to PR negative (78% vs 58%, *P* < .001) and ki67 < 10% compared to ≥10% (92% vs 63%, *P* < .001). The intensity of ER and the presence of perineural or lymphovascular invasion had no significant impact on concordance.

**Conclusions:**

Compared to PREDICT, using OncotypeDx in node negative, ER positive disease is expected to change the clinical decision in a quarter of patients. The concordance between OncotypeDx and PREDICT is influenced by pathological features. In patients with very low risk, treatment decisions may be made based solely on clinical risk assessment.

## Graphical abstract

The concordance in treatment decision making for adjuvant chemotherapy based on OncotypeDX and PREDICT tool is unknown. In this real‐world cohort study that included 445 women with node negative, estrogen receptor positive and HER2 negative disease, the concordance between OncotypeDX and PREDICT was 75%. Concordance was significantly influenced by pathological features with relatively high concordance in women with clinical low risk disease, including tumor size ≤ 1 cm, grade 1 or ki67 < 10%, suggesting using OncotypeDX provides no benefit over using PREDICT alone in very low risk disease.

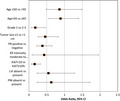

## 1 INTRODUCTION

Breast cancer is the most common cancer in women, and most patients are diagnosed with early‐stage disease.^1^ While adjuvant chemotherapy should be considered in all fit patients,^2^ in many low‐risk patients with hormone receptor positive, human epidermal growth factor receptor 2 negative (HER2) disease, the toxicity from chemotherapy may out‐weigh the potential benefit, therefore identifying these patients is desired.

Tumor and patient characteristics have an important role in treatment decisions.^3^, ^4^, ^5^, ^6^ The development of several genomic assays, such as OncotypeDX (Genomic Health) and MammaPrint (Agendia), have introduced the potential role of genomic risk assessment in treatment decision‐making.^7^, ^8^, ^9^, ^10^ OncotypeDX is one of the more commonly used commercial assays and the first to be recommended by the NICE and ASCO guidelines.^10^ Based on an assay of 21 genes, a recurrence score (RS) that ranges from 0 to 100 is both prognostic for recurrence and predictive for chemotherapy benefit in early‐stage ER positive, HER2 negative disease.^11^, ^12^ Several retrospective studies have identified that a RS higher than 30 indicates high‐risk disease^13^ and more recently the TAILORx study established that RS ≤ 25 is an appropriate threshold for chemotherapy omission.^3^ In this prospective study, women with node negative disease and RS between 11 and 25 had no benefit from adjuvant chemotherapy. Of note, in subgroup analysis, women aged 50 or less had a modest benefit from chemotherapy when RS was between 16 and 25,^7^ however, this benefit is most likely related to chemotherapy associated premature ovarian suppression rather than actual benefit from chemotherapy.^14^

Genomic assays add additional information that may change treatment decisions^9^, ^15^, ^16^, ^17^ but they also incur a high cost and delay treatment. PREDICT is a modern online prognostication tool, that estimates the absolute benefit of systemic treatment on overall‐survival (OS) following breast cancer surgery.^18^ Based on clinical outcome data of several large cancer registries, PREDICT provides data for the average expected benefit from treatment options.^19^, ^20^, ^21^ The advantages of this tool are that there is no delay to decision‐making, and there is no additional financial cost. These data have an important role in physician‐patient decision‐making and since the implementation of PREDICT in 2011 there has been a steady increase in its use all over the world, reaching over 20,000 accesses per month in October 2016.^22^

It remains unclear whether genomic tests should be used for all patients with node negative, ER positive, and HER2 negative disease. According to the updated ASCO guidelines, MammaPrint should not be used in clinically low‐risk patients,^10^ as these patients have an excellent prognosis regardless of the genomic risk.^9^ A recent analysis from the TAILORx has shown significant difference in outcome between high and low clinical risks, regardless to the RS.^22^ These data further emphasize the independent role of clinical risk assessment in estimating the actual benefit from chemotherapy. In this study, we aimed to identify the concordance in treatment decision‐making on adjuvant chemotherapy based on OncotypeDX and on PREDICT in a real‐world cohort. We also aimed to identify pathological and clinical characteristics that have an impact on the concordance rate in order to better recognize patients that their treatment decisions could be done based only on clinical risk assessment.

## 2 METHODS

We performed a retrospective single‐center cohort study. The study cohort included all women who were treated in our institute for hormone receptor positive, HER2 negative, node negative breast cancer diagnosed between 4/2005 and 3/2012, whose tumor tissue was sent for OncotypeDX analysis. The following patients were excluded: men, node positive disease, HER2 positive, or hormone receptor negative. Patients with missing data to calculate the benefit from chemotherapy by PREDICT (such as grade or tumor size) were also excluded.

The patients' medical records were reviewed and prespecified data on patient clinical parameters were extracted, including: age, menopausal status, and mode of detection. Additionally, histo‐pathological characteristics were extracted including: tumor size, nodal involvement, the intensity of ER and progesterone receptor (PR), grade, lymphovascular and perineural invasion and Ki67. Patients' data were anonymized and deidentified prior to analysis. As third generation chemotherapy (such regimens comprising of dose dense anthracyclines and taxanes) is usually recommended for patients with higher risk disease such as node positive or ER negative disease), we calculated the estimated 10‐year OS improvement from second generation chemotherapy using the PREDICT 2.1v tool.^18^ The study protocol was approved by the ethics committee in our institution.

Expected recommendation for adjuvant chemotherapy was assessed by both RS and PREDICT. RS higher than 25 was considered as high genomic risk and RS 25 or lower was considered as low genomic risk. Omission of chemotherapy was expected for low genomic risk^7^ or when the improvement in 10‐year OS by PREDICT was lower than 2%. The 2% threshold was chosen based on a prior survey evaluating patients’ choices of adjuvant chemotherapy according to expected benefit^23^ and based on the authors’ experience, estimating that improving 10‐year OS by 2% or higher will justify the potential long‐term risks associated with adjuvant chemotherapy. The tests were considered concordant for women with RS ≤ 25 and estimated PREDICT benefit < 2% or for women with RS > 25 and estimated PREDICT benefit ≥ 2%. According to the TAILORx study in women aged 50 or younger a potential modest benefit from chemotherapy was seen when RS was ≥16 which was even more prominent when RS ≥ 21.^7^ Therefore, in younger women concordance was also assessed when utilizing RS < 16 or RS < 21 for chemotherapy omission. The influence on concordance of prespecified histological characteristics was assessed including: tumor size, intensity of ER (strong to moderate vs weak expression) and PR (positive vs negative), grade (grade 1 vs grade 2‐3), Ki67 (<10% vs ≥10%) and perineural and lymphovascular invasion (present vs absent). The impact of age on concordance was also assessed utilizing two thresholds: age ≤ 50 vs >50 and age ≥65 vs <65.

### 2.1 Statistical analysis

The statistical analysis was preformed using SAS Software, Version 9.4. Continuous variables were depicted by mean values ± standard deviation, categorical variables were presented by (N %). Concordance was presented using percentages and the kappa coefficient (K). *T* test was used to compare the value of continuous variables between study groups and chi‐squared (for more than two groups) or Fisher's exact tests (for two groups) were used to compare the value of categorical variables between study groups. The difference between the subgroups was presented with odds ratio (ORs) and 95% confidence intervals. Two‐sided *P*‐values less than .05 were considered statistically significant.

## 3 RESULTS

**FIGURE 1:**
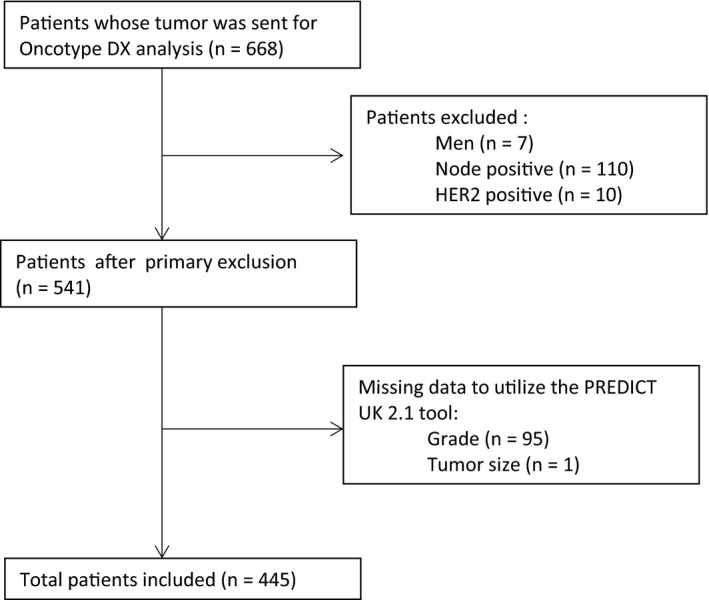
Patients selection

Between 4/2005 and 3/2012, OncotypeDX test was performed for 686 patients in our institution. After exclusions, 445 women were included (see Figure 1). Patients’ characteristics and the differences in the characteristics by the genomic risk are detailed in Table 1. Women with high genomic risk were more likely to have larger tumors (*P* = .008), lower intensity of ER staining (*P* < .001), negative PR (*P* < .001), higher grade (*P* < .001), and higher ki67 (*P* < .001). Additionally, they were significantly more likely to have higher benefit from chemotherapy based on PREDICT results (*P* < .001).

**TABLE 1:** Characteristics of the included patients

Characteristic	All cohort (n = 445)	Recurrence score 0‐25 (n = 347)	Recurrence score > 25 (n = 98)	*P* value*
Median age (range)	60 (34‐85)	60 (34‐85)	59 (35‐83)	.988
Age ≤ 50 year [num (%)]	89 (20%)	65 (19%)	24 (24%)	.252
Premenopausal^a^	94 (22%)	72 (22%)	22 (23%)	.778
Postmenopausal	332 (78%)	260 (78%)	72 (77%)	
Detected by screening^a^	367 (85%)	286 (84%)	81 (86%)	.747
Detected by symptoms	66 (15%)	53 (16%)	13 (14%)	
Tumor size				
Median (IQ)	1.5 (0.3‐5.0)	1.5 (0.3‐5.0)	1.7 (0.5‐3.5)	.008
Mean (SD)	1.59 (0.65)	1.55 (0.65)	1.74 (0.62)	
T ≤ 1 cm	106 (24%)	91 (26%)	15 (15%)	.008
1 < T≤2 cm	254 (57%)	199 (57%)	199 (57%)	
T > 2 cm	85 (19%)	57 (17%)	28 (29%)	
Grade 1	75 (16%)	70 (20%)	5 (5%)	<.001
Grade 2	291 (65%)	237 (68%)	54 (55%)	
Grade 3	79 (19%)	40 (12%)	39 (40%)	
Intensity of ER expression				
Mean (SD)	2.47 (0.55)	2.55 (0.48)	2.21 (0.71)	<.001
Intensity of ER expression				
ER > 2	333 (75%)	273 (78%)	60 (61%)	<.001
2 ≥ ER >1	105 (24%)	72 (21%)	33 (34%)	
ER ≤ 1	7 (1%)	2 (1%)	5 (5%)	
Intensity of PR expression				
Mean (SD)	1.44 (1.06)	1.61 (1.02)	0.81 (0.95)	<.001
PR negative–num (%)	69 (16%)	34 (10%)	35 (36%)	
Ki67%<10%^a^	109 (34%)	103 (41%)	6 (8%)	<.001
Ki67 ≥ 10%	213 (66%)	147 (59%)	66 (92%)	
LVI absent^a^	403 (94%)	316 (95%)	87 (92%)	.265
LVI present	30 (6%)	17 (5%)	8 (8%)	
PNI absent^a^	410 (96%)	319 (96%)	91 (96%)	1.0
PNI present	18 (4%)	14 (4%)	4 (4%)	
Estimated 10‐year OS improvement from second‐generation chemotherapy ≥ 2%^b^	98 (22%)	55 (16%)	43 (44%)	<.001

Abbreviations: ER, Estrogen receptor; IQ, Interquartile range; LVI, Lymphovascular invasion; OS, Overall‐survival; PNI, Perineural invasion; PR, Progesterone receptor; SD, Standard deviation; T, Tumor size.

^a^ Data were not available for: detection mode n = 12, menopausal status n = 19, Ki67 n = 123, LVI n = 17, PNI n = 17.

^b^ The improvement from chemotherapy was calculated utilizing the PREDICT UK 2.1 tool.

^*^ *P* value for the difference between low RS to high RS.

Overall, using PREDICT, the estimated 10‐year improvement in OS from second generation chemotherapy was expected to be low, with 0%‐1% improvement for 347 (78%) women, 2% for 71 (16%) women and 3%‐4% for 27 (6%) women. Chemotherapy was expected to be recommended in 98 (22%) women based on both RS (using threshold of 25) and PREDICT (when estimating 10‐year OS improvement ≥2%). However, overall there was poor concordance between these two tools (*K* = 0.283). A total of 55 women out of 347 (16%) with low benefit by PREDICT were expected to be recommended for chemotherapy based on RS and 55 women out of 98 (56%) with high benefit by PREDICT were expected to be recommended to omit chemotherapy based on RS (see Table 2).

**TABLE 2:** Concordance of physician decision based on PREDICT and Oncotype DX RS

	Estimated 10‐year OS improvement by PREDICT			Concordance rate^a^	Kappa Coefficient
	<2%	≥ 2%			
All (n = 445)					
Oncotype RS ≤ 25 (n = 347)	292 (66%)	55 (12%)		76%	0.284
Oncotype RS > 25 (n = 98)	55 (12%)	43 (10%)			
Age ≤ 50 (n = 89)					
Oncotype RS < 21 (n = 47)	44 (50%)	3 (3%)		67%	0.303
Oncotype RS ≥ 21 (n = 42)	27 (30%)	15 (17%)			
Age ≤ 50 (n = 89)					
Oncotype RS < 16 (n = 22)	22 (25%)		0	45%	0.158
Oncotype RS ≥ 16 (n = 67)	49 (55%)		18 (20%)		
Age ≤ 50 (n = 89)					
Oncotype RS ≤ 25 (n = 65)	58 (65%)		7 (8%)	77%	0.381
Oncotype RS > 25 (n = 24)	13 (15%)		11 (12%)		
Age > 50 (n = 356)					
Oncotype RS ≤ 25 (n = 282)	234 (66%)		48 (13%)	75%	0.255
Oncotype RS > 25 (n = 74)	42 (12%)		32 (9%)		

^a^ Concordance was considered when either RS ≤ 25 and the estimated by PREDICT is < 2% or when RS > 25 and the estimate by PREDICT is ≥ 2%.

**FIGURE 2:**
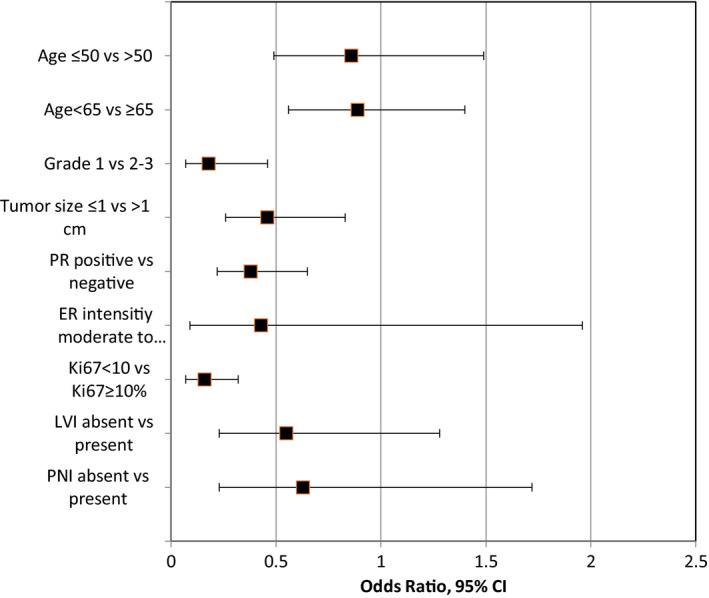
Impact of histo‐pathological variables on concordance

The concordance between PREDICT and RS according to prespecified characteristics is shown in Figure 2 and Table 3. Elaboration of results by type of expected recommendation (ie, chemotherapy vs omission of chemotherapy) by RS and by PREDICT is shown in supplementary Table 1. Grade, tumor size, expression of PR, and ki67% had statistically significant impact on the concordance rate. The other evaluated characteristics, including intensity of ER expression, lymphovascular, and perineural invasion, had no impact on concordance rate. The high concordance rates for grade 1 disease (93%), for ki67 < 10% (92%) or for tumors size ≤1 cm (85%) were driven by low RS for the vast majority of these patients, which was consistent with estimated low benefit by PREDICT. The low concordance rate (51%) for grade 3 disease was mostly driven by patients with low RS and high benefit by PREDICT. Women with tumors larger than 2 cm were associated with relatively low concordance rate (67%) which was also driven mostly by low RS and high benefit by PREDICT. The low concordance (57%) for patients without PR expression was mostly driven by high RS and estimated low benefit by PREDICT.

**TABLE 3:** Impact of age and histological characteristics on the concordance between oncotype and predict tool

	Estimated 10‐year OS improvement by PREDICT		Concordance rate^a^	Subgroups difference—*P* value
	<2%, n (%)	≥2%, n (%)		
Age ≤ 50 (n = 89)				
Oncotype RS ≤ 25 (n = 65)	58 (65%)	7 (8%)	78%	Age ≤50 vs >50 0.584
Oncotype RS > 25 (n = 24)	13 (15%)	11 (12%)		
Age > 50 (n = 356)				
Oncotype RS ≤ 25 (n = 282)	234 (66%)	48 (13%)	75%	
Oncotype RS > 25 (n = 74)	42 (12%)	32 (9%)		
Age < 65 (n = 304)				
Oncotype RS ≤ 25 (n = 238)	202 (66%)	36 (12%)	76%	Age < 65 vs ≥65 0.617
Oncotype RS > 25 (n = 66)	37 (12%)	29 (10%)		
Age ≥ 65 (n = 141)				
Oncotype RS ≤ 25 (n = 109)	90 (64%)	19 (13%)	74%	
Oncotype RS > 25 (n = 32)	18 (13%)	14 (10%)		
Grade 1 (n = 75)				
Oncotype RS ≤ 25 (n = 70)	70 (93%)	0	93%	Grade 1 vs grade 2‐3: <0.001
Oncotype RS > 25 (n = 5)	5 (7%)	0		
Grade 2 (n = 291)				
Oncotype RS ≤ 25 (n = 237)	219 (76%)	18 (6%)	78%	
Oncotype RS > 25 (n = 54)	48 (16%)	6 (2%)		
Grade 3 (n = 79)				
Oncotype RS ≤ 25 (n = 40)	3 (4%)	37 (47%)	51%	
Oncotype RS > 25 (n = 39)	2 (3%)	37 (47%)		
Tumor size ≤ 1 cm (n = 106)				
Oncotype RS ≤ 25 (n = 91)	89 (84%)	2 (2%)	85%	T ≤ 1 cm vs T > 1 cm 0.009
Oncotype RS > 25 (n = 15)	14 (13%)	1 (1%)		
Tumor size > 1 and ≤ 2cm (n = 253)				
Oncotype RS ≤ 25 (n = 199)	167 (66%)	33 (13%)	74%	
Oncotype RS > 25 (n = 54)	33 (13%)	21 (8%)		
Tumor size > 2 cm (n = 85)				
Oncotype RS ≤ 25 (n = 57)	36 (42%)	21 (25%)	67%	
Oncotype RS > 25 (n = 28)	7 (8%)	21 (25%)		
Strong ER expression (n = 333)				
Oncotype RS ≤ 25 (n = 273)	225 (68%)	48 (14%)	77%	ER strong‐moderate vs weak: 0.263
Oncotype RS > 25 (n = 60)	31 (9%)	29 (9%)		
Moderate ER expression (n = 105)				
Oncotype RS ≤ 25 (n = 72)	65 (62%)	7 (7%)	73%	
Oncotype RS > 25 (n = 33)	21 (20%)	12 (11%)		
Weak ER expression (n = 7)				
Oncotype RS ≤ 25 (n = 2)	2 (29%)	0	58%	
Oncotype RS > 25 (n = 5)	3 (42%)	2 (29%)		
PR expression positive (n = 375)				
Oncotype RS ≤ 25 (n = 313)	265 (71%)	48 (13%)	80%	PR positive vs negative: <0.001
Oncotype RS > 25 (n = 62)	32 (8%)	30 (8%)		
PR expression negative (n = 69)				
Oncotype RS ≤ 25 (n = 34)	27 (39%)	7 (10%)	58%	
Oncotype RS > 25 (n = 35)	22 (32%)	13 (19%)		
Ki67 < 10% (n = 109)				
Oncotype RS ≤ 25 (n = 103)	100 (92%)	3 (3%)	92%	Ki67 < 10 vs ≥10% <0.001
Oncotype RS > 25 (n = 6)	6 (5%)	0		
Ki67 ≥ 10% (n = 213)				
Oncotype RS ≤ 25 (n = 147)	105 (49%)	42 (20%)	63%	
Oncotype RS > 25 (n = 66)	36 (17%)	30 (14%)		
Ki67 unknown (n = 123)				
Oncotype RS ≤ 25 (n = 58)	87 (71%)	10 (8%)	82%	
Oncotype RS > 25 (n = 47)	13 (11%)	13 (11%)		
LVI present (n = 25)				
Oncotype RS ≤ 25 (n = 17)	12 (48%)	5 (20%)	64%	LVI present vs absent: 0.161
Oncotype RS > 25 (n = 8)	4 (16%)	4 (16%)		
LVI absent (n = 403)				
Oncotype RS ≤ 25 (n = 316)	271 (67%)	45 (11%)	76%	
Oncotype RS > 25 (n = 87)	50 (13%)	37 (9%)		
LVI unknown (n = 17)				
Oncotype RS ≤ 25 (n = 14)	9 (53%)	5 (29%)	65%	
Oncotype RS > 25 (n = 3)	1 (6%)	2 (12%)		
PNI present (n = 18)				
Oncotype RS ≤ 25 (n = 14)	11 (61%)	3 (17%)	66%	PNI present vs absent: 0.363
Oncotype RS > 25 (n = 4)	3 (17%)	1 (5%)		
PNI absent (n = 410)				
Oncotype RS ≤ 25 (n = 319)	272 (66%)	47 (12%)	76%	
Oncotype RS > 25 (n = 91)	51 (12%)	40 (10%)		
PNI unknown (n = 17)				
Oncotype RS ≤ 25 (n = 14)	9 (53%)	5 (29%)	65%	
Oncotype RS > 25 (n = 3)	1 (6%)	2 (12%)		

Abbreviations: ER, Estrogen receptor; LVI, Lymphovascular invasion; PNI, Perineural invasion; PR, Progesterone receptor.

^a^ Concordance was considered when either oncotype RS ≤ 25 and the estimated by PREDICT is <2 or when RS > 25 and the estimate by PREDICT is ≥2.

Eighty‐nine (20%) women were aged 50 or younger. The concordance rates when considering lower RS threshold for chemotherapy recommendation in this subgroup are shown in Table 2. For threshold or RS ≥ 21 the concordance was similar to the concordance in all patients, but when considering a lower threshold of RS 16, the concordance between PREDICT and RS was worse (44.9%, *K* = 0.158). This was driven by a low benefit according to PREDICT together with RS 16 or higher for the majority of the younger women. In contrast, when the improvement in 10‐year OS by PREDICT was 2% or higher, the RS was also 16 or higher for all women in this subgroup (see Table 2).

## 4 DISCUSSION

Treatment for early‐stage breast cancer has evolved remarkably during recent decades, resulting in a significant improvement in outcomes.^2^, ^24^, ^25^, ^26^ Adjuvant chemotherapy has a potential to improve survival in early‐stage breast cancer patients,^2^ however, it is associated with short‐ and long‐term toxicity. Therefore, identifying patients with potential clinically meaningful benefit from adjuvant chemotherapy is crucial. Early stage ER positive, HER2 negative disease is known to have the lowest absolute benefit from chemotherapy compared to the other breast cancer subtypes,^27^ and multigene signatures may be useful to optimize treatment decisions.^10^

Unrestricted use of genomic tests, however, may lead to a considerable economic burden and delay treatment decisions. Therefore, identification of populations whose treatment decision is unlikely to be influenced by genomic assays could have an important economic impact and speed up decision‐making. Our results of high rates of concordance for women with very low clinical risk, including women with tumors 1 cm or smaller, with grade 1 disease or with ki67 < 10% suggest that OncotypeDX in these patients is unlikely to change treatment decision and therefore could be avoided. These findings are consistent with the conclusions of a recent systematic review on cost‐effectiveness analyses of OncotypeDX, suggesting OncotypeDX is cost‐effective for women with clinically intermediate‐ or high‐risk disease, but not for the women with clinically low‐risk disease.^28^ Omission of chemotherapy without genomic assessment in clinically low‐risk women in further supported in the results of the MINDACT study showing chemotherapy had no effect in women with low clinical risk and high genomic risk.^9^

Studies evaluating the cost‐effectiveness of genomic signatures have shown inconsistent results. While the UK National Institute for Health and Care Excellence (NICE) considers these tests to be cost‐effective for intermediate risk of recurrence based on clinico‐pathological characteristics,^29^, ^30^ other economic analyses have concluded that OncotypeDX is cost‐effective for a much larger group.^15^, ^16^, ^17^, ^31^, ^32^ Of note, some of the cost‐effectiveness analyses have several important limitations and methodological concerns: in almost all studies the real‐world distribution of RS was unreliable as some models used the NSABP B‐14 results in which information on HER2 was not available, adverse events related to chemotherapy were often ignored in the models and available risk classification models such as Adjuvant! Online or PREDICT were used only in the minority of the cost‐effectiveness studies and most studies did not analyze the cost‐effectiveness by clinical risk.^28^

We found that grade, tumor size, ki67, and expression of PR had a statistically significant impact on concordance rate. Aside from PR expression, all of these variables are included in PREDICT.^18^ PR expression is a well‐known prognostic characteristic in breast cancer.^6^ In light of our results, we believe further investigation to evaluate the role of PR expression in quantifying the benefit from chemotherapy should be considered, as it may better estimate the clinical risk based on the available immunohistochemical characteristics.

This study has several limitations. First, as this is a single center study and data were extracted retrospectively, it is vulnerable to unknown bias. Second, real‐world decisions are made after discussing risk and benefit with the patient, however, in this study, we determined an arbitrary threshold for chemotherapy recommendation. Third, data on comorbidities were not taken into consideration, in contrast to real‐world decision‐making. However, it is reasonable to assume that patients, whose physicians opt to send for OncotypeDX analysis, are fit enough to receive chemotherapy and have reasonable life expectancy. Last, while genomic risk was assessed by OncotypeDX, other genomic signatures are also used and there could be a discordance between OncotypeDX and the other signatures.

In conclusion, compared to PREDICT use of OncotypeDX in node negative, ER positive, HER2 negative breast cancer, is expected to change treatment decisions in a quarter of the patients. As the concordance between PREDICT and OncotypeDX is influenced by pathological features and is much higher in clinically very low‐risk disease, the added value of OncotypeDX in these patients is questionable and it is not clear whether the associated budget impact and the delay in treatment decisions justify its use in such patients.

## CONFLICT OF INTEREST

Moore declared *honorarium* payment from MSD. Sarfaty declared *honorarium* payment from Roche, Novartis, and MSD. Yerushalmi declared consulting fee from Roche, Pfizer, and Novartis. Invited speaker from Roche, Teva, Medison, MSD, Astra‐Zeneca, and Novartis. Goldvaser declared *honorarium* payment from Roche, Pfizer, Novartis, Oncotest. The other authors have no conflict of interest to declare.

## AUTHOR CONTRIBUTIONS

Daniel Goldstein was involved in conceptualization, methodology, writing—original draft, and writing—review and editing. Chen Mayer, Michal Sarfaty, and Rinat Yerushalmi were involved in investigation, resources, and writing—review and editing. Tzippy Shochat was involved in formal analysis and writing—review and editing. Daniel Reinhorn and Assaf Moore were involved in investigation and writing—review and editing. Hadar Goldvaser was involved in conceptualization, methodology, investigation project administration, supervision, writing—original draft, and writing—review and editing.

All authors approved the final version of the manuscript and agree to be accountable for aspects of the work.

## Data Availability

The data that support the findings of this study are available on request from the corresponding author. The data are not publicly available due to privacy or ethical restrictions.
